# Hidden transmissions of *Pseudomonas aeruginosa* ST111 –the importance of continuous molecular surveillance

**DOI:** 10.1186/s13756-025-01619-1

**Published:** 2025-08-15

**Authors:** Jasmin Kaur Jasuja, Eva-Maria Klupp, Martin Aepfelbacher, Knut Kurt William Kampe, Michael Fabian Nentwich, Stefan Kluge, Johannes Karl-Mark Knobloch

**Affiliations:** 1 Department for Infection Prevention and Control, Institute for Microbiology, Virology and Hygiene, University Medical Center Hamburg-Eppendorf, Martinistraße 52, 20246 Hamburg, Germany; 2 Institute for Medical Microbiology, Virology and Hygiene, Institute for Microbiology, Virology and Hygiene, University Medical Center Hamburg-Eppendorf, Martinistraße 52, 20246 Hamburg, Germany; 3 Department of Intensive Care, University Medical Center Hamburg-Eppendorf, Martinistraße 52, 20246 Hamburg, Germany

**Keywords:** Outbreak, High-risk clone, *Pseudomonas aeruginosa*, ST111, Whole genome sequencing, *bla*_VIM−2_

## Abstract

**Background:**

A series of transmission of *Pseudomonas aeruginosa* ST111 *bla*_VIM−2_, previously undetected by standard surveillance, was discovered in a tertiary care hospital in Northern Germany through molecular genetic monitoring. Hence, environmental sampling was initiated to find the source of infection.

**Methods:**

First, routine epidemiological data ruled out patient-to-patient transmission and two initial diagnoses were assessed as externally acquired. After the discovery of the highly related cluster by whole genome sequencing, a more detailed epidemiological analysis was carried out, including previous hospitalizations. An environmental investigation was initiated due to a possible connection of transmissions with an intensive care unit.

**Results:**

Between 2018 and 2023 16 clinical isolates of *Pseudomonas aeruginosa* ST111 *bla*_VIM−2_ were identified of which 12 isolates belonged to ST111 carrying an In59-like integron. Routine whole-genome sequencing of carbapenem resistant *P. aeruginosa* identified a highly related cluster (maximum of three allelic differences) of high-risk ST111 isolates in ICU patients over five years, confirming sink-to-patient transmission associated to sink drains in two ICU rooms. In initial routine epidemiological categorization of these highly related isolates four isolates were categorized as possible nosocomial acquisition without direct epidemiological link to other patients, whereas two isolates were categorized as ‘externally acquired’.

**Conclusions:**

This finding highlights the ability of high-risk clone ST111 to persist in hospital environments and emphasizes the importance of integrating molecular surveillance with routine epidemiology to uncover hidden transmissions. In this case, the frequent detection of the ST111 high-risk clone led to targeted environmental sampling, uncovering a prolonged outbreak that had gone unnoticed by conventional surveillance. The clone was eliminated from the ward during a reconstruction project.

**Supplementary Information:**

The online version contains supplementary material available at 10.1186/s13756-025-01619-1.

## Background

*Pseudomonas aeruginosa* is a leading cause of healthcare-associated infections (HAIs) and is of particular concern in critical care settings. As an important cause of healthcare-acquired infections in intensive care units (ICUs), *P. aeruginosa* demonstrates a remarkable ability to rapidly develop resistance to various antimicrobials, driven by chromosomal mutations and the acquisition of resistance genes encoded on mobile genetic elements such as plasmids or transposons [1–6]. The Centres for Disease Control and Prevention (CDC) and the European Centre for Disease Prevention and Control (ECDC) define multidrug resistance (MDR) as co-resistance to at least one agent in three out of eight antimicrobial categories [7].

MDR *P. aeruginosa*, including *P. aeruginosa* clones harbouring plasmid-encoded carbapenemases, are prevalent globally and typically observed in hospital environments. These clones are selected within hospitals due to antibiotic selection pressure and favourable growth conditions [8]. Thereby, in *P. aeruginosa* isolates spontaneously developing phenotypic resistance by mutations during antibiotic therapy must be distinguished from isolates carrying specific resistance genes like carbapenemases [9]. The emergence of widely disseminated carbapenemase producing *P. aeruginosa* (CP-PA) strains, designated as high-risk clones, has made whole-genome sequencing (WGS) an essential tool for understanding their epidemiology. Several high-risk clones are identified by their sequence types (STs) and have emerged on an international scale. Most hospital outbreaks in Europe are associated with multi locus sequence types (MLST) ST111, ST175, ST233, ST235, ST277, ST357, ST654 and ST773 [2, 8, 10]. CP-PA strains are frequently detected in wastewater and rinse water, where they form multi-bacterial biofilms in plumbing systems [11]. Within these protective and impenetrable biofilms, Enterobacterales and *P. aeruginosa* strains exchange carbapenemase-encoding plasmids [11]. During outbreaks, clones associated with the aforementioned STs are often found causing infections in vulnerable patient populations within ICUs. In a systematic review by Büchler et al. 100 out of 126 included studies screened the environment in outbreak situations and in all but three contaminated environment was identified as the primary source [12]. Hence, environmental screening was identified as an important outbreak control measure [12].

Here, we report a hidden outbreak with metallo-β-lactamase *bla*_VIM−2_-producing and *qacE*∆1-harbouring *P. aeruginosa* ST111 in a German tertiary care hospital, uncovered by perennial routine core genome multilocus sequencing typing (cgMLST), involving sink-to-patient transmission. To identify the outbreak source, an epidemiological investigation was initiated. Environmental samples were collected from the relevant ICUs, and the identified *P. aeruginosa* strains were analysed using cgMLST.

## Results

### Outbreak description

**Fig. 1:**
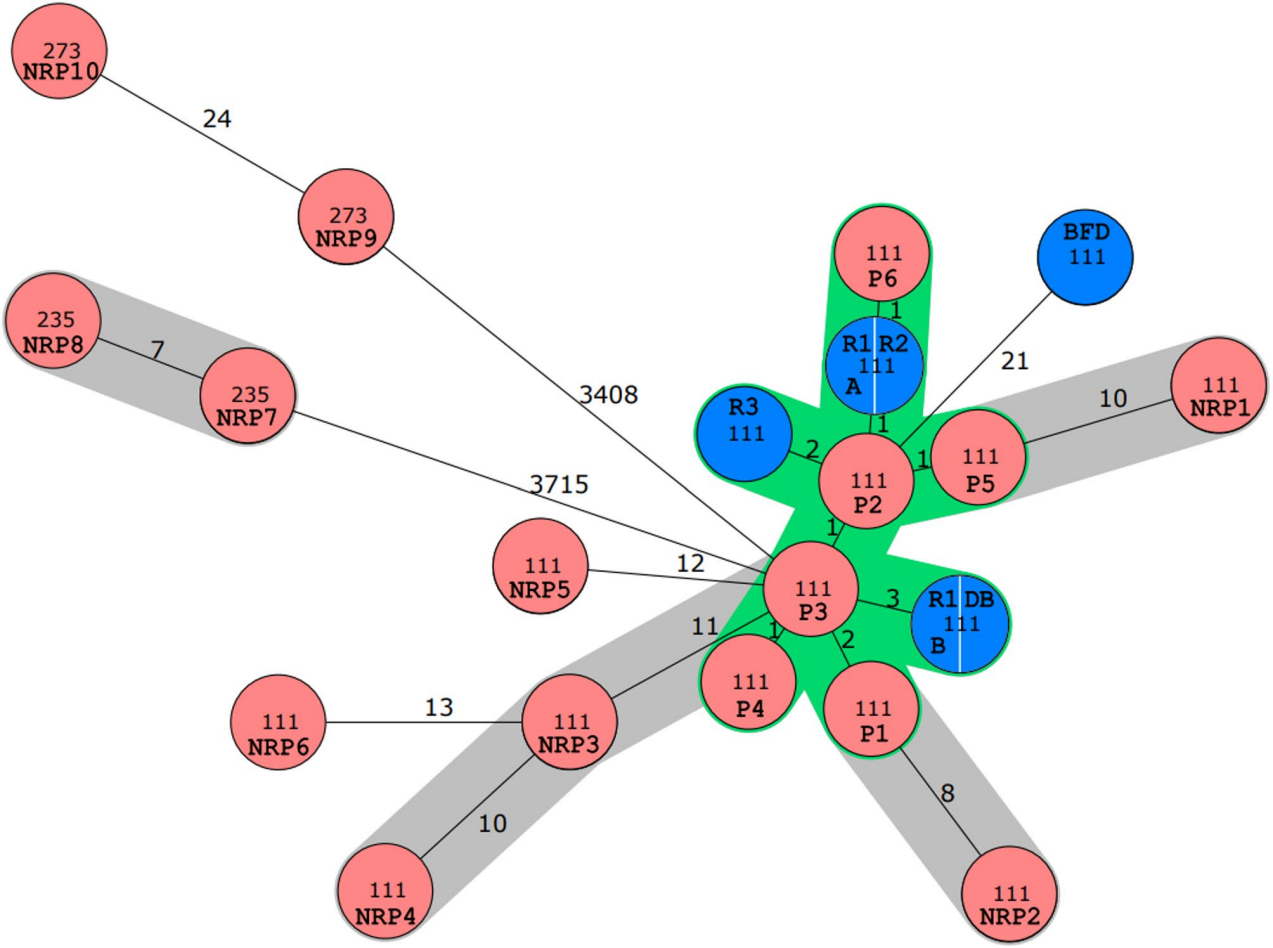
Minimum spanning tree of *bla*_VIM-*2*_-positive *Pseudomonas aeruginosa* of a tertiary care hospital in northern Germany. A total of 16 *Pseudomonas aeruginosa bla*_VIM-2_ clinical isolates (red isolates, P1-6: patient 1 to 6; NRP1-10: non-related patient 1 to 10), were identified by WGS in clinical isolates from July 2018 to August 2023 and were analysed by cgMLST. A total of 4478 core genes were used. The grey shading marks clusters with the published threshold for clonal relatedness (≤ 11 distinct alleles). Within, a highly related cluster of ≤ 3 distinct alleles (green shading) six patient isolates (P1-P6) clustered with five environmental samples (blue isolates R1 = room 1; R2 = room 2; R3 = room 3; DB = dialysis bag; BFD = bedpan flushing device)

Between July 2018 and August 2023, a total of 131 non-repetitive phenotypically carbapenem-resistant *P. aeruginosa* (CR-PA) isolates cultured from clinical specimens of an adult hospital were analysed by cgMLST. Among these, carriage of carbapenemase genes were identified in 29 isolates (22.1%), thereof 16 isolates harbouring the *bla*_VIM−2_ gene (55.2%), four isolates harbouring *bla*_VIM−1_ (13.8%), and two isolates harbouring *bla*_VIM−5_ (6.9%). The predominant ST was ST111 (*n* = 14), followed by ST273 (*n* = 5). cgMLST identified a cluster (≤ 11 different alleles) of eleven ST111 CP-PA *bla*_VIM−2_ isolates, all also harbouring *qacE*∆1, a gene encoding an efflux pump for quaternary ammonium compounds (QAC). Six out of eleven *bla*_VIM−2_*P. aeruginosa* ST111 isolates were identified as highly related with a maximum allelic distance of three alleles (Fig. 1). In initial routine epidemiological categorization of these closely related isolates four isolates were categorized as possible nosocomial acquisition without direct epidemiological link to other patients, whereas two isolates were categorized as ‘externally acquired’ as patients were re-admitted or hospitalized after external stays. Due to the confirmed highly close relationship of the individual isolates but first recovery months and even years apart a more detailed epidemiological re-investigation was initiated, focusing on these isolates.

### Epidemiology

**Fig. 2:**
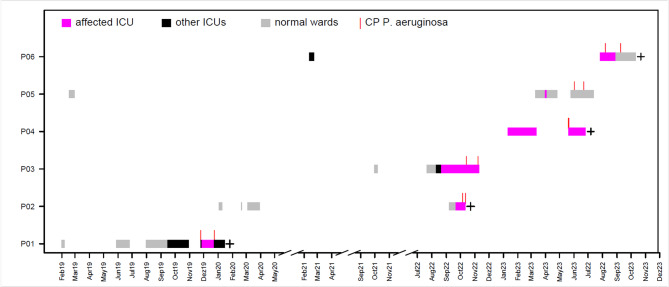
Line list of patients with confirmed transmission events of ST111 Pseudomonas aeruginosa bla_VIM-2_. A total of six patients were affected (P1-P6). The broad horizontal lines mark the respective stays in a normal ward (grey) of the affected intensive care unit (magenta) or another intensive care unit in the hospital. The vertical red lines indicate the respective first and last detection of a CP-PA in the affected patients. The black cross marks patients, who died in the course of their hospitalization

The dates of first detection of the six highly related isolates were ranged between December 2019 and August 2023. Initial microbiological detections were made from relevant clinical specimens including the respiratory tract, skin, wounds and blood cultures (Fig. 2). An initial screening at admission was only conducted in two out of six patients, proving colonization with CP-PA. Hence, most of these cases were classified as nosocomial according to the German hospital infections surveillance system (KISS). The first externally acquired case was first hospitalized early 2019 and had an infection 298 days after the initial admission with intermittent hospitalization. The second externally acquired case was admitted early 2023 and infection with the outbreak strain was detected 130 days later with intermittent hospital stays. The initial detection in nosocomial-acquired cases occurred 8, 11, 29 and 85 days post admission (median: 20 days), respectively, and were all found in clinical specimen. Retrospective analysis showed that all six patients had infections caused by *P. aeruginosa* ST111 *bla*_VIM−2_, sourced from various clinical conditions, such as surgical site infections, acute respiratory distress syndrome (ARDS), pneumogenic sepsis, pneumonia and anastomosis- and respiratory insufficiency. Of these, four patients died in the further course of their hospital stay. Whether the ST111 infection was the cause of death cannot be ruled out with certainty.

We subsequently investigated the wards where the initial detections occurred, and it was found that four of the six detections were from ward A in the years 2022 and 2023, with the remaining two from wards B in 2019 and D in 2023, respectively.

Since these findings did not immediately point to a clear source for possible transmissions, previous hospital stays were reviewed, particularly as the affected patients had been hospitalised for extended periods. The review indicated that the isolates detected on wards B and D were also associated with prior stays on ward A (Fig. 2). Interestingly, all affected patients could be associated with a stay in two adjacent patient rooms on ward A. As a result, environmental sampling was initiated on ward A.

### Environmental sampling and molecular epidemiology

The extensive environmental examinations (see methods) were conducted twice in November 2023 and comprised 36 environmental samples (15 specimen from sinks of adjacent bathroom across seven rooms, six specimen from wardrobes in the bathrooms, one medical product kept in one of the patient room, two from a toilet in a patient room and twelve specimen from a sluice room) (Supplementary Fig. 1). The wastewater piping of rooms R1 and R2 are connected horizontally to the same wastewater line. Room R3 is separately connected to a wastewater pipeline, which merges only with the line serving R1 and R2 in the basement. During environmental sampling no positive screened patient was occupying the affected rooms and ward, respectively. Eleven environmental CR-PA isolates were identified. Thereof, two isolates were non-carbapenemase producing *P. aeruginosa*. In total, nine CP-PA isolates were identified with *bla*_VIM−2_ (*n* = 5) being the predominant carbapenemase. MLST by whole genome sequencing uncovered that all environmental *bla*_VIM−2_*P. aeruginosa* belonged to ST111. *P. aeruginosa* ST111 *bla*_*VIM−2*_ was found in three out of six sinks in patient rooms, on the surface of a dialysis therapy bag kept near the washbasin in patient’s bathroom, and in the sink of a bedpan flushing device in the sluice room. Applying a threshold for high relation of ≤ 3 different alleles, we identified five environmental *bla*_VIM−2_ and *qacE*∆1 positive *P. aeruginosa* ST111 isolates clustering with clinical isolates of patients hospitalised between 2019 and 2023. The four environmental samples were obtained from three washbasin sinks and from the exterior of a dialysis bag stored above a washbasin. All four environmental *P. aeruginosa* ST111 *bla*_VIM−2_ isolates were found in three patient rooms (R1-R3). Thereof, all patients had an associated stay for rooms R1 and R2, but none for R3. One specimen from the sluice room was positive for ST111 but did not harbour any carbapenemase and cluster distance threshold was > 12.

In order to assess whether plasmid transfer was involved in the transmission events, a detailed analysis of the resistance gene profile was carried out and attempts were made to determine the location of *bla*_VIM−2_. The resistance gene profile was almost identical between the ST111 isolates, including identical resistance-associated mutations and a profile of genes associated with reduced sensitivity to biocides and heavy metals (Supplementary Table, orange section). Analysis of the immediate genomic environment of *bla*_VIM−2_ revealed for ten of the ST111 isolates contigs so small (930 to 1539 bases) that it was not possible to evaluate their location. For eight isolates, assembly resulted in larger contigs (15 to 50 kb). The highly related isolates showed homology to chromosomally integrated In59-like class 1 integrons (Supplementary Fig. 2; Supplementary Table green section), which carry the resistance genes *aac*(6’)-29, *qacE*∆1and *sul*1 in addition to *bla*_VIM−2_ [13]. However, de novo assembly revealed an In59-like integron without *bla*_VIM−2_ in some strains, as well as some possible insertions and deletions in this region (Supplementary Fig. 2; Supplementary Table green section). This observation may also be due to the limitations of short read sequencing. As screening for plasmid replicon sequences using PlasmidFinder resulted in no hits for known plasmids in the database chromosomal integration of *bla*_VIM−2_ is suggested.

Due to the positive environmental findings, re-sampling of the environment was initially planned. However, from April to May 2024 the ward was closed for renovation and conversion into a regular ward. During this time, no patients were admitted to these rooms and consequently washbasins were not in use. Microbiological controls after reopening the ward did not yield additional *P. aeruginosa* ST111 *bla*_VIM−2_ isolates in six investigations over a period of twelve months.

## Discussion

In this investigation, we uncovered a hidden prolonged outbreak involving the high-risk clone *P. aeruginosa* ST111 *bla*_*VIM−2*_ in a hospital in northern Germany, which had not been recognised as an outbreak by routine epidemiological surveillance. A purely epidemiological assessment was hindered by its prolonged nature over five years going along with patient transfers, external stays, and re-admissions with colonization or infection possibly being misclassified as externally acquired. Hence, epidemiological assessment alone carries the risk to detect only a fraction of the actual transmissions. The frequent occurrence of *P. aeruginosa* ST111 *bla*_*VIM−2*_ isolates, defined as highly related cluster in molecular surveillance, prompted a closer examination of its epidemiology. From an epidemiological perspective, patient-to-patient transmission was ruled out as there was no direct contact between these patients, all of whom had been hospitalised in ward A but during different periods of time. The investigation of further common procedures like dialyses and bronchoscopy were also excluded, as not every aforementioned patient was dialysed or got a bronchoscopy, especially prior to infection with *P. aeruginosa* ST111 *bla*_*VIM−2*_. However, all patients were associated with a stay in two adjacent patient rooms in ward A. As a result of this retrospective epidemiological analysis, repetitive environmental sampling was performed on ward A.

The observation of allelic differences far less than the distance threshold for genomic relatedness of ≤ 11 different in five environmental and clinical isolates of *P. aeruginosa* ST111 *bla*_*VIM−2*_ proved a nearly identical clone and confirmed sink-to-patient transmission for these cases [14, 15]. Indeed, sink colonisation by MDR Gram-negative bacteria is well documented and is considered as a potential transmission route, frequently associated with HAIs and posing a risk even in non-outbreak situations [16, 17]. Efforts to replace or eradicate contaminated sinks have been employed, but often fail [18], and it is even recommended that ICU rooms should not be equipped with sinks [19]. Our findings align with this, as the environmental *P. aeruginosa* ST111 *bla*_*VIM−2*_ strain remained detectable in 2023, despite its initial molecular detection occurring four years earlier. However, only six *P. aeruginosa* ST111 *bla*_*VIM−2*_ infections were traced back to specific sinks in the ICU, though it is assumed that ST111 persists throughout the year in patients [20]. If the infections with ST111 were indeed hospital-acquired, the initial classification of some cases as ‘externally acquired’ under the KISS definition would no longer apply. However, the environmental samplings showed ten different *P. aeruginosa* ST111 *bla*_VIM−2_ isolates indicating the overall frequency of sink-to-patient transmission was low but increased with the length of hospital stay as R1 and R2 was often occupied by patients with long-term stays, while patients in R3 did not. However, Rath et al. analysed the toilet-to-patient transmission rate of ST235 *bla*_FIM−1_ and ST309 non-carbapenemase producing *P. aeruginosa* in a bone marrow transplant unit with cgMLST, showing only a low-genetic diversity but only three toilet-to-patient-transmission over six years [21]. Indeed, our reported ward was originally built as an intermediate care unit and was converted into an intensive care unit later on. As a result, the washbasins were not located directly in the patient’s room but in a separate bathroom, which might have reduced the frequency of transmission.

In addition, alternative transmission routes must be considered. The positive screening of a dialysis therapy bag, placed above the washbasin suggests a possible transmission route, though not all patients were receiving dialysis. Rather, the dialysis therapy bag may represent a potential sink-to- environment-transmission, which might have been favoured by different pressure conditions leading to sink-to-environment-transmission. Bronchoscopy was excluded as a common transmission route as well as other environmental sources on epidemiological basis. However, it should be noted that environmental investigation was initiated four years after the first detection of *P. aeruginosa* ST111 *bla*_*VIM−2*_ and hence, potential transmission routes might have been missed meanwhile.

Interestingly, the first *P. aeruginosa* ST111 *bla*_*VIM−2*_ isolate was already detected in 2019 in a patient in screening and blood culture, which was conducted on admission to ward A and was declared as externally acquired since the patient was transferred from an external hospital where the patient was treated for one month. Before that, the patient had hospital stays in our hospital, but remained on normal wards. A *P. aeruginosa* was never detected. All other cases in the cluster occurred after the admission of the index case bringing up the discussion that first the outbreak stem was transmitted from patient to sink following hidden sink-to-patient transmission and leading to the hidden outbreak. We found a median duration of 20 days from admission to infection with the outbreak strain, which is lower than reported by Volling et al. [22]. However, Volling et al. reported a non-ST111 *P. aeruginosa* outbreak strain. In the two externally acquired cases colonisation time a maximum of 298 days and 130 days, respectively, is suggested. Notably, during this period both patients were not continuously hospitalized, making it hard to estimate the colonization-to-infection-period.

In our study, the *qacE*Δ1 resistance gene was detected in ST111 isolates as part of the In59-type integron, similar to the findings of Rath et al. However, it remains unclear whether this gene contributes to resistance against QAC-based disinfectants or plays a role in outbreak promotion even if a QAC-based disinfectant was used in routine hospital disinfection.

ST111 as well as other ST’s multi-resistance is well-documented, but association to resistance gene *qacE*∆1 has not been reported so far. Hence, further research is needed to elucidate any direct connections between this clone and *qacE*Δ1.

The observation of *P. aeruginosa* ST111 *bla*_*VIM−2*_ isolates with confirmed sink-to-patient transmission in an ICU-setting highlights the vulnerability of ICU patients and the conducive environment for antibiotic selection pressure. Previous studies have similarly documented the predominance of ST111 in ICUs and other high-risk wards, including in Germany, where ST111 *bla*_*VIM−2*_ was identified in a cluster of 15 isolates as well as in a Greek hospital [14, 21, 23]. ST111 strains are frequently associated with carbapenemase production, particularly *bla*_*VIM−2*_, leading to multidrug resistance [24, 25]. By chance, the transmission series might be interrupted by eliminating of the clone from the ward during a reconstruction project.

### Limitations

This study has several limitations. First, environmental sampling was conducted four years after the first detection of the high-risk clone in clinical isolates. As a result, earlier environmental isolates and sources may have been missed, limiting our ability to accurately determine the duration of the high-risk clone’s presence in the hospital environment in- and outside the sink. To address this, we propose regular environmental sampling to allow for the timely identification of high-risk clones.

Second, while all patients included in the study were infected with the high-risk clone, only two out of six patients were initially screened for colonisation. Therefore, it is unclear whether transmission of the high-risk clone frequently leads to direct infection or if infection regularly resulted from previously undetected colonisation. Hence, we suggested that long-term patients should be screened during their ICU stay. However, further investigation is needed to determine the required time from colonisation to infection, as well as the trigger factors transforming a colonisation into an infection.

Third, during de novo assembly, *bla*_VIM−2_ was located in a chromosomally integrated In59-like integron in some strains. In strains without detectable integration into In59, it is unclear whether this is an artefact of the assembly or whether genetic events have actually taken place. However, the three resistance genes *bla*_VIM−2_, *qacE*Δ1 and *sul*1 were detected in all ST111 strains. Current literature as well as our detailed analysis of the resistance gene profile does not provide sufficient data directly linking *qacE*Δ1 to ST111 *P. aeruginosa* and the role in hospital transmission and infection. Further research is required to elucidate these questions.

Fourthly, the outbreak took place during the pandemic, which led to a change in the usual patient clientele.

## Conclusion

Our report highlights the importance of molecular surveillance, which is more sensitive in detecting high-risk clones compared to conventional epidemiological assessment, which is often hampered by clinical processes such as internal and external transfers and prolonged hospital stays. Epidemiological surveillance should account for several years of data, particularly in cases of high genomic relatedness, to detect silent transmissions at an early stage. Colonisations and infections initially classified as externally acquired may, upon retrospective analysis, prove to be hospital-acquired, leading to the misclassification of silent transmissions over several years, as observed in our case. In this context, it is particularly important that medical institutions that conduct such detailed epidemiological analyses are not blamed for the discovery of transmission events that would have gone unnoticed in institutions without targeted molecular and epidemiological analyses.

## Methods

The investigation was conducted from July 2018 to August 2023 in a tertiary care hospital in northern Germany, encompassing all medical specialities excluding paediatrics. During this period, all *P. aeruginosa* clinical isolates of adult wards recovered from routine diagnostic except screening material were collected. Isolates exhibiting phenotypic resistance to piperacillin, ceftazidime, ciprofloxacin, meropenem and imipenem based on the official multidrug-resistant (MDR) definition by the German healthcare authorities underwent further molecular sequencing.

For DNA extraction, the QIASymphony SP Instrument and QIAsymphony DSP Virus/Pathogen Mini Kit (Qiagen, Venlo, The Netherlands) were used as recently published [26]. Sequencing was performed using the NextSeq500 platform (Illumina, San Diego, USA), in combination with the NEBNext Ultra DNA Library Prep Kit and NEBNext Multiplex Oligos for Illumina (NEB, Ipswich, USA). Assembly of genomes and cgMLST was conducted with the Seqsphere software package (Ridom, Münster, Germany Version 11.0.0; integrated Velvet assembler Version 1.1.04), applying a clustering threshold of ≤ 11 allelic differences, based on literature review [14, 21]. A total of 4478 core genes were used for cgMLST. Detailed information about the assembled genomes is given in the Supplementary Table (blue section). The assembled genomes of the six patients, the non-related patients and environmental specimen have been deposited to the public database (NCBI GeneBank database) under the BioProject no. PRNJA1288733. Resistance gene analysis was conducted using the AMRFinder (Version 1.3.1) integrated in the Seqsphere package (all identified genes or mutations displayed in the Supplementary Table, orange section). Additionally, screening for plasmid replicon sequences was performed using PlasmidFinder 2.1 [27]. Large contigs containing *bla*_VIM−2_ were analysed for homologies to known sequences using nucleotide BLAST (https://blast.ncbi.nlm.nih.gov/Blast.cgi). After identifying an In59 integron (accession number AF263519.1) as closely related, an alignment of all contigs with a length of more than 10,000 base pairs containing *bla*_VIM−2_ and/or *qacE*∆1 in comparison to the In59 integron and the *bla*_VIM−2_ gene alone (Supplementary Fig. 2) was generated using MAUVE (version 20150226) and the progressiveMauve alginment [28].

Following epidemiological indications as aforementioned, we conducted an environmental sampling in the ICU ward with association to the ST111 cluster. The environmental sampling was initiated in November 2023 with a focus on sinks and rinse water. Sinks in six bathrooms and surrounding of the washbasin across seven rooms and the unclean work space were sampled. A total of 36 specimen were collected in two rounds of environmental sampling.

For sampling, swabs and moistened sponge were utilised for siphons and environmental surroundings, respectively, transported to the microbiology laboratory, and processed within 12 h. Swabs were vortexed and subcultured onto blood agar, MacConkey agar, and ESBL agar (bioMérieux). Sponges were packed in sterilised boxes and were incubated in Tryptic Soy Broth (TSB) at 37 °C for 24 h. Afterwards 10 µl were subcultured on ESBL agar and incubated at 36 °C under aerobic conditions for 24–48 h. Colonies consistent with *P. aeruginosa* subcultured from swabs and sponges were identified via MALDI-TOF MS, and antimicrobial susceptibility testing was performed with VITEK2. If phenotypic MDR *P. aeruginosa* was detected, aforementioned sequencing protocol was applied to the environmental isolates.

Sink-to-patient transmission was defined as hospital-acquired colonisation (detected in rectal swabs, throat swabs, clinical specimen without initiating antibiotic treatment) or infection (clinical specimen with initiation of antibiotic treatment) with *P. aeruginosa* ST111 *bla*_*VIM−2*_ with a distance threshold of ≤ 11 alleles to ST111 *bla*_*VIM−2*_ isolates previously recovered from the environment in a room occupied by the respective patient for at least 72 h.

## Supplementary Information

Below is the link to the electronic supplementary material.

Supplementary Material 1

Supplementary Material 2

Supplementary Material 3

## Data Availability

No datasets were generated or analysed during the current study.

## Abbreviations

ARDS: Acute respiratory distress syndrome
CDC: Centres for Disease Control and Prevention
CgMLST: Core genome multilocus sequencing typing
CP-PA: Carbapenemase producing *P. aeruginosa*
CR-PA: Carbapenem-resistant *P. aeruginosa*
DTR: Difficult-to-treat resistant
ECDC: European Centre for Disease Prevention and Control
HAIs: healthcare-associated infections
ICUs: Intensive care units
KISS: Hospital infections surveillance system
MDR: Multidrug resistance
MLST: Multi locus sequence types
*P. aeruginosa*: *Pseudomonas aeruginosa*
QAC: Quaternary ammonium compounds
STs: Sequence types
TSB: Tryptic Soy Broth
WGS: Whole-genome sequencing

## References

[1] Lambert ML, Suetens C, Savey A, Palomar M, Hiesmayr M, Morales I, et al. Clinical outcomes of health-care-associated infections and antimicrobial resistance in patients admitted to European intensive-care units: a cohort study. Lancet Infect Dis. 2011;11(1):30–8. 21126917 10.1016/S1473-3099(10)70258-9

[2] de Almeida de Souza GH, Rossato L, Brito GT, dos Santos Bet GM, Simionatto S. Carbapenem-resistant *Pseudomonas aeruginosa* strains: a worrying health problem in intensive care units. Rev Inst Med Trop Sao Paulo. 2021;63:e71. 34586305 10.1590/S1678-9946202163071 PMC8494492

[3] Gleyce Hellen de Almeida de Souza, Rossato L, Brito GT. Graciela mendonça Dos Santos bet, Simone simionatto. Carbapenem-resistant Pseudomonas aeruginosa strains: a worrying health problem in intensive care units. Rev Inst Med Trop Sao Paulo. Sep 2021;27:63–71. 10.1590/S1678-9946202163071 PMC8494492 34586305

[4] Moradali MF, Ghods S, Rehm BH. *Pseudomonas aeruginosa* lifestyle: a paradigm for adaptation, survival, and persistence. Front Cell Infect Microbiol. 2017;7:39. 28261568 10.3389/fcimb.2017.00039 PMC5310132

[5] Breidenstein EB, de la Fuente-Núñez C, Hancock RE. *Pseudomonas aeruginosa*: all roads lead to resistance. Trends Microbiol. 2011;19(8):419–26. 21664819 10.1016/j.tim.2011.04.005

[6] Potron A, Poirel L, Nordmann P. Emerging broad-spectrum resistance in *Pseudomonas aeruginosa* and A*cinetobacter baumannii*: mechanisms and epidemiology. Int J Antimicrob Agents. 2015;45(6):568–85. 25857949 10.1016/j.ijantimicag.2015.03.001

[7] Magiorakos AP, Srinivasan A, Carey RB, Carmeli Y, Falagas ME, Giske CG, et al. Multidrug-resistant, extensively drug-resistant and pandrug-resistant bacteria: an international expert proposal for interim standard definitions for acquired resistance. Clin Microbiol Infect. 2012;18(3):268–81. 21793988 10.1111/j.1469-0691.2011.03570.x

[8] Kocsis B, Szabó D. Diversity and distribution of resistance markers in *Pseudomonas aeruginosa* international high-risk clones. Microorganisms. 2021;9(2):359. 33673029 10.3390/microorganisms9020359 PMC7918723

[9] Yoon EJ, Goussard S, Touchon M, Krizova L, Cerqueira G, Murphy C, et al. Mobile carbapenemase genes in *Pseudomonas aeruginosa* reveal complex transmission dynamics. Antimicrob Agents Chemother. 2021;65(3):e01440–20.

[10] Oliver et al. The increasing threat of Pseudomonas aeruginosa high-risk clones. Drug Resist Updates July-August 2015, pp. 41–59. 10.1016/j.drup.2015.08.002 26304792

[11] Weingarten RA, Johnson RC, Conlan S, Ramsburg AM, Dekker JP, Lau AF, Khil P, Odom RT, Deming C, Park M, Thomas PJ, NISC Comparative Sequencing Program, Henderson DK, Palmore TN, Segre JA, Frank KM. mBio. 2018;9(1):e02011–17. 10.1128/mBio.02011-17. PMID: 29437920; PMCID: PMC5801463. Genomic Analysis of Hospital Plumbing Reveals Diverse Reservoir of Bacterial Plasmids Conferring Carbapenem Resistance. 10.1128/mBio.02011-17 PMC5801463 29437920

[12] Büchler AC, Heudorf U, Kirchner M, Gölz H, Kempf VAJ, Haller S. Outbreak investigations after identifying carbapenem-resistant *Pseudomonas aeruginosa*: a systematic review. Antimicrob Resist Infect Control. 2023;12(1):32. 37013661 10.1186/s13756-023-01223-1 PMC10068724

[13] Poirel L, Lambert T, Türkoglü S, Ronco E, Gaillard J, Nordmann P. Characterization of class 1 integrons from *Pseudomonas aeruginosa* that contain the *bla*_VIM-2_ carbapenem-hydrolyzing β-lactamase gene and two novel aminoglycoside resistance gene cassettes. Antimicrob Agents Chemother. 2001;45(2):546–52. 10.1128/AAC.45.2.546-552.2001. 11158753 10.1128/AAC.45.2.546-552.2001 PMC90325

[14] Wendel AF, Kolbe-Busch S, Ressina S, Schulz C, Buhl T, Wendt C, et al. Genomic-based transmission analysis of carbapenem-resistant *Pseudomonas aeruginosa* at a tertiary care centre in Cologne (Germany) from 2015 to 2020. JAC Antimicrob Resist. 2022;4(2):dlac047. 35611260 10.1093/jacamr/dlac057 PMC9122648

[15] Rath S, Klein S, Wasner M, Bletz S, Schleicher T, Autenrieth IB, et al. Whole-genome sequencing reveals two prolonged simultaneous outbreaks involving *Pseudomonas aeruginosa* high-risk strains ST111 and ST235 with resistance to quaternary ammonium compounds. J Hosp Infect. 2024;135:155–64. 10.1016/j.jhin.2024.01.009 38286239

[16] Fucini C, Kieffer N, Bletz S, Klein S, Schleicher T, Autenrieth IB, et al. Sinks in patient rooms in ICUs are associated with higher rates of hospital-acquired infection: a retrospective analysis of 552 ICUs. J Hosp Infect. 2023;137:99–105. 10.1016/j.jhin.2023.05.018 37308060

[17] Hopman J, Tostmann A, Wertheim H, Bos M, Voss A. Reduced rate of intensive care unit acquired Gram-negative bacilli after removal of sinks and introduction of ‘water-free’ patient care. Antimicrob Resist Infect Control. 2017;6:59. 28616203 10.1186/s13756-017-0213-0 PMC5466749

[18] Pirzadian J, ‘t Voor. Holt AF, Hossain M, Klaassen CHW, de Goeij I, Koene HHHT, Bode LGM, Vos MC, Severin JA. Limiting spread of VIM-positive *Pseudomonas aeruginosa* from colonized sink drains in a tertiary care hospital: a before-and-after study. *PLoS One*. 2023;18(3):e0283346. 10.1371/journal.pone.0282090 PMC10038242 36961784

[19] Catho G, Martischang R, Boroli F, Mouton W, Schrenzel J, Harbarth S. Outbreak of *Pseudomonas aeruginosa* producing VIM carbapenemase in an intensive care unit and its termination by implementation of waterless patient care. Crit Care. 2021;25(1):302. 34412676 10.1186/s13054-021-03726-y PMC8376114

[20] De Greyter S, De Smet D, Deplano A, Fournier D, Denis O. Sink drains as reservoirs of VIM-2 metallo-β-lactamase-producing *Pseudomonas aeruginosa* in a Belgian intensive care unit: relation to patients investigated by whole-genome sequencing. J Hosp Infect. 2021;115:75–82. 34111433 10.1016/j.jhin.2021.05.010

[21] Rath S, Bletz S, Autenrieth IB, Mutters NT. Retrospective genome-oriented analysis reveals low transmission rate of multidrug-resistant *Pseudomonas aeruginosa* from contaminated toilets at a bone marrow transplant unit. J Hosp Infect. 2024;137:96–104. 10.1016/j.jhin.2024.05.015 38830540

[22] Volling C, Mataseje L, Graña-Miraglia L, Hu X, Anceva-Sami S, Coleman BL, Downing M, Hota S, Jamal AJ, Johnstone J, Katz K, Leis JA, Li A, Mahesh V, Melano R, Muller M, Nayani S, Patel S, Paterson A, Pejkovska M, Ricciuto D, Sultana A, Vikulova T, Zhong Z, McGeer A, Guttman DS, Mulvey MR. Epidemiology of healthcare-associated *Pseudomonas aeruginosa* in intensive care units: are sink drains to blame? J Hosp Infect. 2024;136:77–86. 10.1016/j.jhin.2024.03.009 38554807

[23] Papagiannitsis CC, Petinaki E, Tzouvelekis LS, Miriagou V, Kontopidou F, Tryfinopoulou K, et al. Unravelling the features of success of VIM-producing ST111 and ST235 *Pseudomonas aeruginosa* in a Greek hospital. Microorganisms. 2020;8(12):1824. 33260774 10.3390/microorganisms8121884 PMC7761518

[24] van der Bij AK, Pitout JD. Metallo-β-lactamase-producing *Pseudomonas aeruginosa* in the netherlands: the nationwide emergence of a single sequence type. Clin Microbiol Infect. 2012;18(9):369–72. 10.1111/j.1469-0691.2012.03969.x 22805614

[25] Witney AA, Gould KA, Pope CF, Bolt F, Stoker NG, Cubbon MD, et al. Genome sequencing and characterization of an extensively drug-resistant sequence type 111 serotype O12 hospital outbreak strain of *Pseudomonas aeruginosa*. Clin Microbiol Infect. 2014;20(10):O609–18. 24422878 10.1111/1469-0691.12528

[26] Carlsen L, Büttner H, Christner M, Franke G, Indenbirken D, Knobling B, Lütgehetmann M, Knobloch J. High burden and diversity of carbapenemase-producing enterobacterales observed in wastewater of a tertiary care hospital in Germany. Int J Hyg Environ Health. 2022;240:113891. 10.1016/j.ijheh.2022.113968 35390565

[27] Carattoli A, Zankari E, García-Fernández A, Voldby Larsen M, Lund O, Villa L, Møller Aarestrup F, Hasman H. In Silico detection and typing of plasmids using plasmidfinder and plasmid multilocus sequence typing. Antimicrob Agents Chemother. 2014;58(7):3895–903. 10.1128/AAC.02412-14. 24777092 10.1128/AAC.02412-14 PMC4068535

[28] Darling AE, Mau B, Perna NT. ProgressiveMauve: multiple genome alignment with gene gain, loss and rearrangement. PLoS ONE. 2010;5(6):e11147. 10.1371/journal.pone.0011147. 20593022 10.1371/journal.pone.0011147 PMC2892488

