# Supplementary figures and images for: Hidden transmissions of Pseudomonas aeruginosa ST111 –the importance of continuous molecular surveillance
Source: Antimicrob Resist Infect Control. 2025 Aug 15;14:99. doi: 10.1186/s13756-025-01619-1 (PMC12357456; doi:10.1186/s13756-025-01619-1)

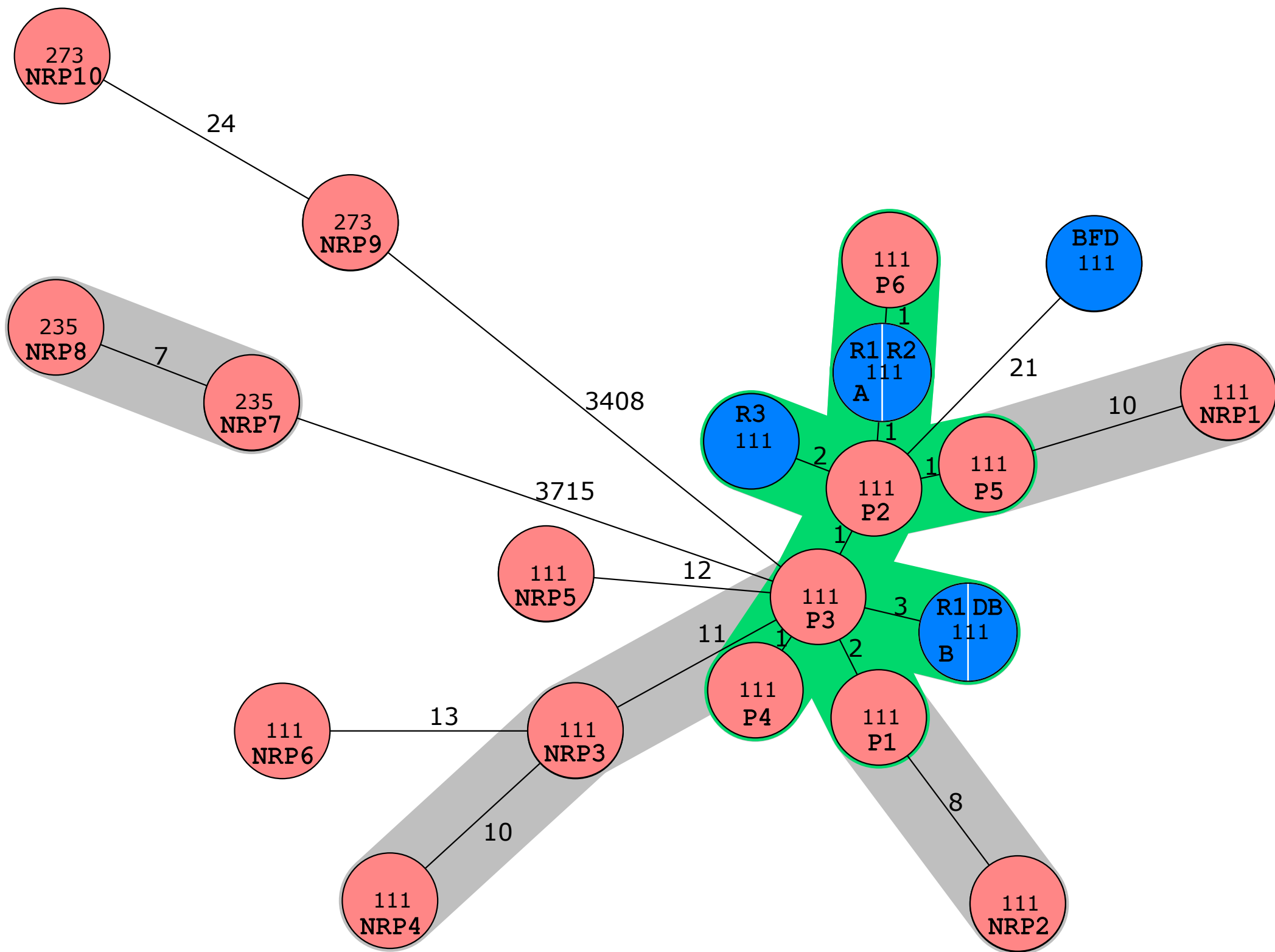

Supplement: Supplementary file 2 — Supplementary Material 2 [file 13756_2025_1619_MOESM2_ESM.pdf]
