# Supplementary material for: Hidden transmissions of Pseudomonas aeruginosa ST111 –the importance of continuous molecular surveillance
Source: Antimicrob Resist Infect Control. 2025 Aug 15;14:99. doi: 10.1186/s13756-025-01619-1 (PMC12357456; doi:10.1186/s13756-025-01619-1)
Supplement: Supplementary file 3 — Supplementary Material 3 [file 13756_2025_1619_MOESM3_ESM.pdf]

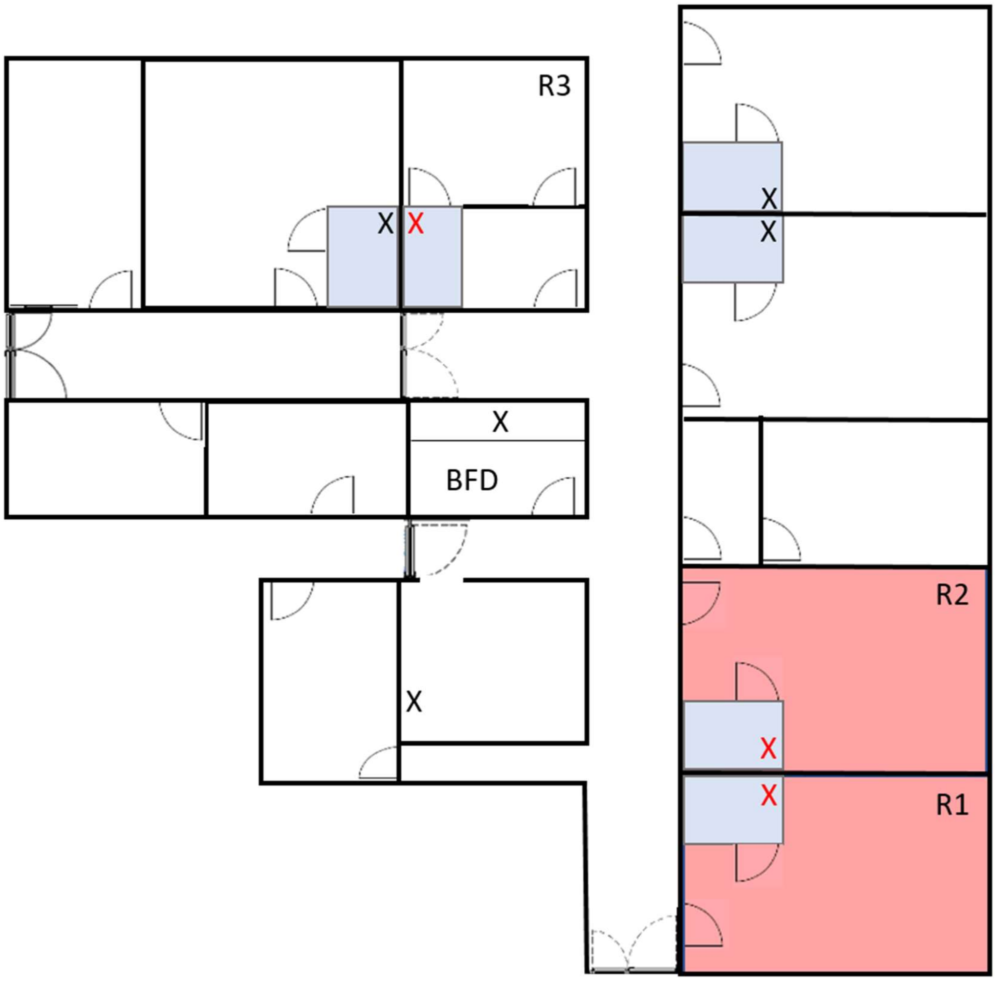

Supplementary Figure 1 *S*chematic floor plan of an outbreak-impacted intensive care unit (ICU) with a series of transmission of Pseudomonas aeruginosa ST111 *bla*$_{\text{VIM-2}}$ from sinks to patients over four years detected by molecular surveillance. Bathrooms are highlighted in blue. Black 'X' symbols denote the presence of siphons, whereas red 'X' symbols indicate siphons with positive environmental test results. Patient rooms with confirmed positive cases are shaded in red (R1 and R2). Siphon in R3 was tested positive but no patient could be associated to the environmental specimen. The abbreviation 'BFD' refers to the bed flushing device, which was tested positive but was not associated to the highly related cluster (≤ 3).
